# CT predicts liver fibrosis: Prospective evaluation of morphology- and attenuation-based quantitative scores in routine portal venous abdominal scans

**DOI:** 10.1371/journal.pone.0199611

**Published:** 2018-07-10

**Authors:** Verena C. Obmann, Nando Mertineit, Annalisa Berzigotti, Christina Marx, Lukas Ebner, Roland Kreis, Peter Vermathen, Johannes T. Heverhagen, Andreas Christe, Adrian T. Huber

**Affiliations:** 1 Department of Diagnostic, Interventional and Pediatric Radiology, Inselspital, Bern University Hospital, University of Bern, Bern, Switzerland; 2 Department of Visceral Surgery and Medicine, Hepatology, Inselspital, Bern University Hospital, University of Bern, Bern, Switzerland; 3 Department for BioMedical Research, Unit for Magnetic Resonance Spectroscopy and Methodology, University of Bern, Bern, Switzerland; Texas A&M University, UNITED STATES

## Abstract

**Objectives:**

Our aim was to prospectively determine whether quantitative computed tomography (CT) scores, consisting of simplified indices for liver remodeling and attenuation, may predict liver fibrosis in abdominal CT scans.

**Materials and methods:**

This cross-sectional, prospective study was approved by the local IRB (Kantonale Ethikkommission Bern). Written informed consent was given from all patients undergoing study-MR exams. Between 02/16 and 05/17, four different liver fibrosis scores (CRL-R = caudate-right-lobe ratio, LIMV-, LIMA- and LIMVA-fibrosis score, with “LIM” for liver imaging morphology, “V” for liver vein diameter and “A” for attenuation) were calculated in 1534 consecutive abdominal CT scans, excluding patients with prior liver surgery and liver metastasis. Patients were invited to undergo magnetic resonance (MR) elastography as the non-invasive gold standard to evaluate liver fibrosis. MR elastography shear modulus ≥2.8 kPa was defined as beginning liver fibrosis, while ≥3.5 kPa was defined as significant liver fibrosis (which would correspond to fibrosis stage F2 or higher in histology). Cutoff values, sensitivities and specificities obtained from the receiver operating characteristics (ROC) analysis were then calculated in 141 patients who followed the invitation for MR elastography. To mitigate selection bias, prevalence was estimated in the screened total population (n = 1534) by applying the cutoff values with sensitivities and specificities calculated in the MR elastography sub-group. Positive predictive values (PPV) and negative predictive values (NPV) were then calculated.

**Results:**

Fibrosis scores including liver vein attenuation LIMA-FS and LIMVA-FS showed higher areas under the ROC curves (0.96–0.97) than CRL-R (0.82) to detect significant liver fibrosis, while LIMV-FS showed good performance as well (0.92). The prevalence-corrected PPV were 29% for CRL-R, 70% for LIMV-FS, 76% for LIMA-FS and 82% for LIMVA-FS.

**Conclusion:**

CT fibrosis scores, notably LIMA-FS and LIMVA-FS, may predict significant liver fibrosis on routine abdominal CT scans.

## Introduction

With demographic trends toward an aging population [1] and with an increasing prevalence of obesity and metabolic syndrome [2], chronic liver disease has become an important healthcare issue [3,4]. Nevertheless, chronic liver disease, and consequent liver fibrosis, remains under recognized [5], leading to significant morbidity and mortality [6]. Indeed, due to its reversibility during treatment [7,8], early diagnosis might decrease the disease burden, prevent hepatocellular carcinoma (HCC) [9] and lower healthcare costs [10].

Several non-invasive imaging methods, especially ultrasound and magnetic resonance (MR) elastography, enable non-invasive detection of liver fibrosis [11]. However, to date no systematic screening program for the general population exists to detect liver fibrosis in clinically occult stages. Cross-sectional studies have shown a significant elevation in liver stiffness (shear modulus ≥8 kPa in FibroScan® assessments, which would correspond to fibrosis grade F2 or higher in histology) in 7.5% of the general population [12], while the prevalence of non-alcoholic fatty liver disease, is now estimated to be as high as 27–42% [13–15]. Current CT methods for detecting liver fibrosis are reader-dependent [16], or they rely on time-consuming image post-processing methods such as liver segmental volumetry [17], CT texture analysis [18] or the liver surface nodularity score [19]. However, quantitative, reproducible metrics to assess liver fibrosis on abdominal CT scans without image post-processing, such as the caudate-right-lobe ratio (CRL-R) [20] and the liver imaging morphology and vein diameter fibrosis score (LIMV-FS) [21], have shown good accuracies to differentiate cirrhotic livers from normal livers with areas under the receiver operating characteristics (ROC) curve of 0.79–0.89. We hypothesized that an extension of these morphology-based fibrosis scores, via a simple comparison of the liver vein attenuation to the inferior vena cava attenuation in the portal venous phase as an indirect and simplified surrogate for liver perfusion (LIMA-FS: liver imaging morphology and attenuation fibrosis score and LIMVA-FS: liver imaging morphology, vein diameter and attenuation fibrosis score), would additionally increase the performance of these scores.

The aim of this study was to prospectively determine whether such quantitative computed tomography (CT) scores may predict liver fibrosis in abdominal CT scans.

## Materials and methods

### Study population

**Fig 1:**
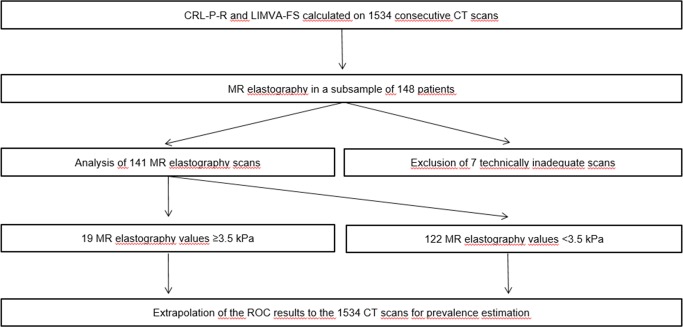
Study workflow chart. CT Fibrosis scores (CRL-R, LIMA-, LIMV- and LIMVA-FS) were calculated on 1534 consecutive CT scans. All patients received invitations for MR elastography, resulting in a subpopulation of 148 following the MR invitation. Optimal cutoffs to predict significant liver fibrosis (≥3.5 kPa in MR elastography) were determined with ROC analysis. These cutoffs with corresponding sensitivities and specificities were then applied to the whole population for prevalence estimation, allowing for calculations of the PPV and NPV of CRL-R, LIMA-, LIMV- and LIMVA-FS to predict significant liver remodeling. CRL-R = caudate-right-lobe ratio, LIMA-FS = liver imaging morphology and attenuation fibrosis score, LIMV-FS = liver imaging morphology and vein diameter fibrosis score, LIMVA-FS = liver imaging morphology, vein diameter and attenuation fibrosis score, CT = computed tomography, MR elastography = magnetic resonance elastography, ROC = Receiver operating characteristics, PPV = positive predictive value, NPV = negative predictive value.

This prospective cross-sectional study was approved by the institutional review board (IRB number 282–15) and conducted after obtaining written patient informed consent. Patients <18 years, patients >70 years and patients with liver masses, portal vein thrombosis, prior liver surgery and contraindications for MR imaging were excluded. A total of 1534 consecutive portal venous phase abdominal CT scans from 1474 patients (52 patients underwent ≥1 CT scan) in our institution were analyzed between 02/2016-05/2017. All these patients were invited for MR elastography as non-invasive reference standard to diagnose liver fibrosis. 148 patients (10%) accepted our invitation without financial compensation. Time interval between CT and MR scans was 116±36 days in average. 7 patients had to be excluded because of technically inadequate MR elastography due to susceptibility artifacts or early cessation of the MR exam due to claustrophobia, resulting in an MR elastography study population of 141 patients (Fig 1). Additionally, 20 consecutive patients with abdominal CT scans and known biopsy-confirmed liver cirrhosis (Metavir F4) were included. To avoid selection bias, these patients were just used as positive controls with 95% confidence intervals but not included in the statistical analysis to determine PPV and NPV of the analyzed fibrosis scores. Clinical information and laboratory test results were recorded.

### CT imaging and post-processing

CT scans were acquired on Siemens Somatom Definition Flash, Definition Edge (Siemens Healthineers, Erlangen, Germany) and Philips Brilliance 64 (Philips, Best, Netherlands) scanners with a pitch of 0.8 and a detector collimation of 0.6. The acquisitions were performed with attenuation based KV selection and automated mAs adaption using references of 100 kVp and 150 mAs. Axial 1-mm slices were reconstructed with an increment of 1 mm in a liver parenchyma window, using the vendor-specific iterative reconstruction algorithm.

Liver remodeling is associated with the following CT morphological changes: 1) atrophy of the right liver lobe and hypertrophy of the caudate and left liver lobe, increasing the CRL-R [20]; 2) compression of the liver veins resulting in a decrease in the liver vein diameter (LVD) [21,22]; and 3) decreased hepatic microperfusion [23,24]. While CRL-R and LVD are represent morphologic parameters as already shown in other studies [20–22,25], we introduced the liver vein to cava attenuation (LVCA) as a simplified surrogate for perfusion. The idea was that mean transit time of IV contrast agent through the liver will be delayed due to altered hepatic perfusion in liver fibrosis [26,27], while the systemic venous blood return will be stable. We hypothesized that the newly introduced liver vein to cava attenuation (LVCA) on portal venous phase would allow a simple comparison of hepatic perfusion time (the time until the contrast has reached the hepatic veins) to the systemic, extrahepatic venous blood return (the time until the contrast has reached the inferior vena cava). In normal liver parenchyma, the applied contrast medium reaches the liver veins earlier than the inferior vena cava. Therefore, a visual categorical grading system depending on the liver vein attenuation compared to attenuation of the inferior vena cava (IVC) was used: 1 hyper-attenuating, 2 iso-attenuating, 3 hypo-attenuation, while a score of 4 was given if liver veins were not visibly contrasted. IVC attenuation was assessed 1 cm inferior to the liver vein confluence and liver vein attenuation was assessed 1 cm proximal to the liver vein confluence. In visually similar attenuation between IVC and liver veins, a region of interest was drawn in the IVC and the largest of the three liver veins, while a difference of less than 20 Hounsfield Units was rated as iso-attenuating.

All 1534 consecutive CT scans were analyzed using four different fibrosis scores (CRL-R, LIMA-, LIMV- and LIMVA-FS) as follows:

$$CRL-R=\frac{CLD}{RLD}$$

$$LIMV-FS=\frac{LVD}{CRL-R}$$

LIMA−FS=CLR−R×LVCA

$$LIMVA-FS=\frac{LVD}{CLR-R\;x\;LVCA}$$

where CLD = caudate lobe diameter, RLD = right lobe diameter, LVD = liver vein diameter, LVCA = liver vein to cava attenuation.

**Fig 2:**
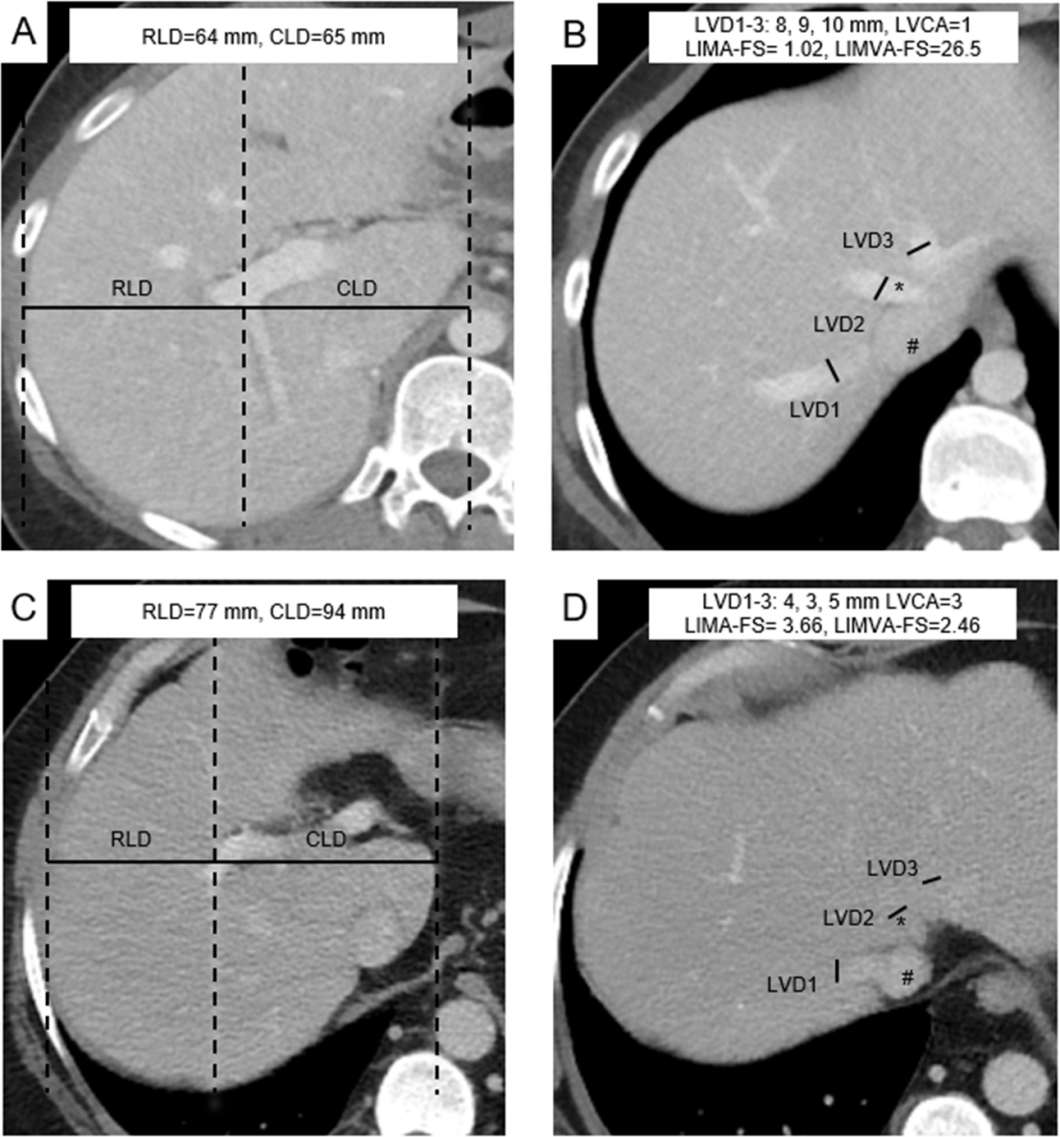
Liver imaging morphology and attenuation fibrosis score (LIMA-FS) and liver imaging morphology, vein diameter and attenuation fibrosis score (LIMVA-FS) in two patients. Images A & C show axial mid-liver slices on the level of the portal vein. The distances of the right lateral border of the right portal vein bifurcation to the lateral margin of the right hepatic lobe (RLD) and to the most medial margin of the caudate lobe (CLD) are measured in an exactly horizontal direction. The two distances were divided CLD/RLD and defined as the caudate-right-lobe ratio (CRL-R). Images B & D show the proximal liver veins and inferior vena cava (IVC). The diameter of each vein is measured, and the sum results in the liver vein diameter (LVD). The liver vein density (*) is compared visually to the ICV density (#), and the liver vein to cava attenuation (LVCA) is assessed. Images A & B are from a 46-year-old female patient without liver remodeling (shear modulus 2.0 kPa) and with LIMA-FS = 1.02 and LIMVA-FS = 26.5. Images C & D are from a 47-year-old male patient with significant liver remodeling (MRE = 4.1 kPa) without steatosis (PDFF = 5.8%). Note that in this patient, the liver veins are not contrasted (LVCA = 4) LIMA-FS = 4.88, LIMVA-FS = 2.46. RLD = right lobe diameter, CLD = caudate lobe diameter, LVD = liver vein diameter, IVC = inferior vena cava, LCVD = liver vein to cava density; LIMA-FS = liver imaging morphology and attenuation fibrosis score; LIMVA-FS = liver imaging morphology, vein diameter and attenuation fibrosis score, PDFF = proton density fat fraction.

**Fig 3:**
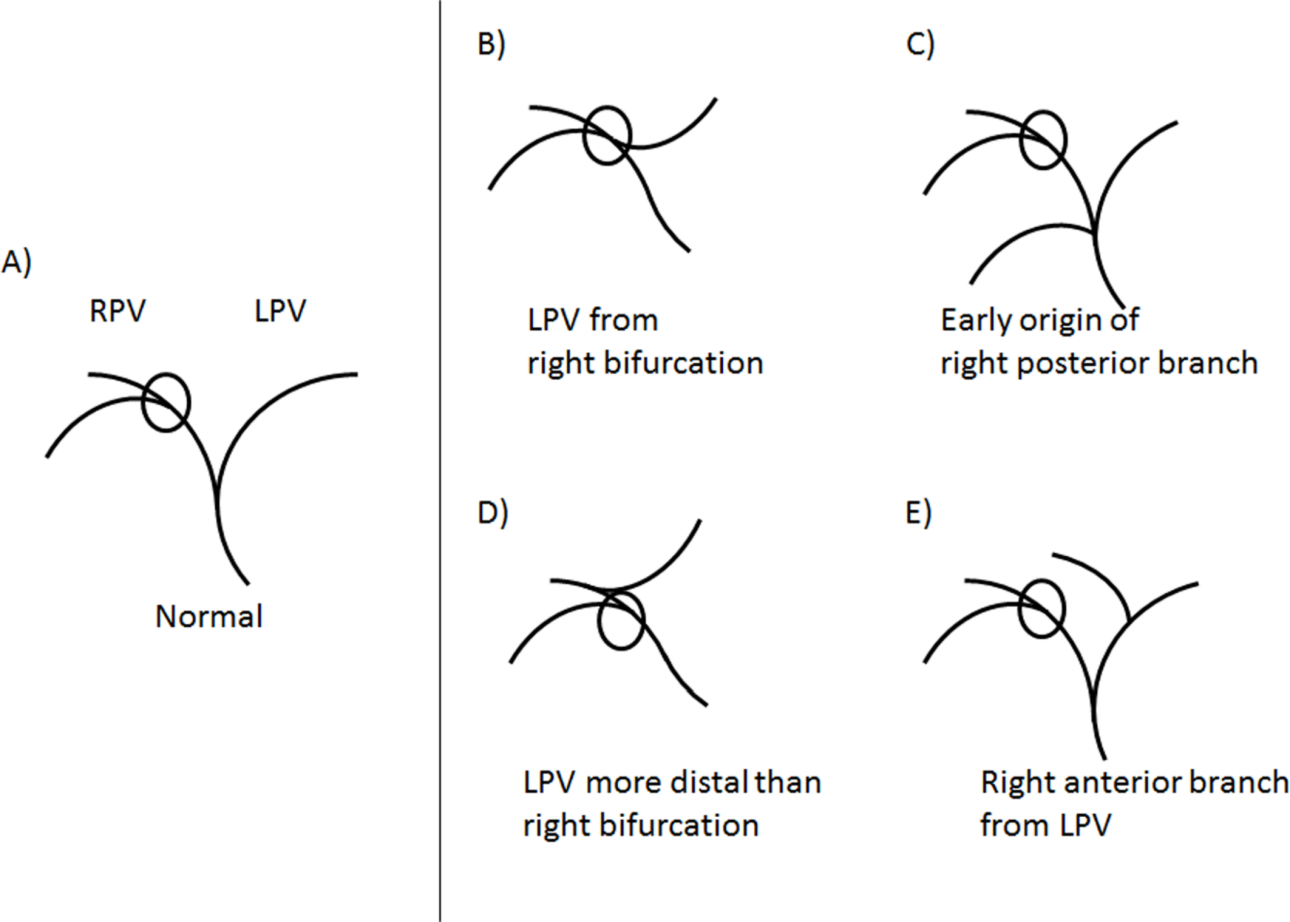
Caudate-right-lobe ratio in case of portal venous variants. In normal anatomy, the first bifurcation of the right portal vein (RPV) is used for the caudate-right-lobe ratio (CRL-R) calculation A), while anatomic variants are shown on the right side of the figure. B-E) In cases of an anomalous origin of the left portal vein (LPV), the first bifurcation of the RPV is used as in the normal variant, illustrated in B) and D). In the case of an early origin of the right posterior branch, the first bifurcation of the right anterior branch is measured C). In the case of an origin of the right anterior branch from the LPV, the first bifurcation of the right posterior branch is measured E). **RPV = right portal vein; CRL-R = caudate-right-lobe-ratio; LPV = left portal vein**.

The medial margin of the caudate lobe and lateral margin of the right liver lobe were identified by scrolling through the slices. The axial section presenting the first right portal vein bifurcation was then selected and the distance to the medial and lateral margin of the right liver (projected on this slice) was measured strictly horizontally from there (Fig 2). These measurements constitute the caudate lobe diameter (CLD) and the right lobe diameter (RLD) used to calculate the CRL-R, as described before [20]. Portal vein anatomy variants were considered as indicated in Fig 3. Liver vein diameter (LVD) was defined as the sum of the three main hepatic vein diameters 1 cm proximal to the aperture into the IVC (Fig 2). Images were evaluated by two radiologists with 3 (N.M.) and 5 (V.O.) years of experience in liver imaging on a dedicated reading workstation with a 5MP display (MDCC-6230, Barco, Kortrijk, Belgium) using Picture Archiving and Communication System (PACS) (IDS7, SECTRA, Linköping, Sweden). In unclear cases, the readers consulted each other’s opinion and then decided in consensus.

### MR imaging technique and imaging analysis

Patients were examined with a 3T-MR system (Verio, Siemens Healthineers, Erlangen, Germany) in a fasting state (>6 h). A pneumatic driver (Resoundant, Rochester, MN, USA) was placed on the right upper quadrant transmitting shear waves by continuous acoustic vibrations at 60 Hz. The liver shear modulus in kPa in the right upper liver lobe was determined with a gradient echo-based elastography sequence (WIP package 622 provided by Siemens Healthineers, 3 single-slice acquisitions with 5-mm slice thicknesses) using the 95% confidence map of stiffness. The MR elastography reading radiologist (A.H.), with 7 years of experience in liver imaging, was blinded to the results of the CT scans. Patients with MR elastography results ≥3.5 kPa were referred to our hepatology department for clinical anamnesis, laboratory tests and FibroScan® assessments. The cutoff value of 3.5 kPa was chosen corresponding to the 8-kPa cutoff in FibroScan®, since it corresponds to a fibrosis stage ≥F2 in histology, generally regarded as significant fibrosis [28–30]. A lower shear modulus cutoff ≥2.8 kPa was considered as beginning liver remodeling (fibrosis stage ≥F1) [31,32]. The Dixon method with axial T1-weighted axial vibe images (TE of 2.45 ms, TR of 5.47 ms, 3-mm slice thickness) was used to calculate the proton density fat fraction to differentiate patients with and without liver steatosis.

### Statistical analysis

Analysis was performed with the statistical software package R version 3.4.1 [33] and GraphPad Prism (Version 7.1, GraphPad Software Inc, CA, USA). Liver shear modulus in kPa wrer compared between different LVCA scores (1–4) using the Mann-Whitney-U test. Pearson correlation was calculated between MR Elastography and LVCA and used to compare CT fibrosis scores with liver shear modulus in kPa. The different CT fibrosis scores and clinical parameters were compared between patients with normal and elevated shear modulus ≥2.8 kPa, as well as between patients with beginning (shear modulus 2.8–3.5 kPa) and significant liver fibrosis (shear modulus ≥3.5 kPa) using the Mann-Whitney-U test for continuous variables and Fisher’s exact test for categorical variables. Optimal cutoff values of the CT fibrosis scores were calculated with the ROC analysis to predict beginning (≥2.8 kPa) and significant (≥3.5 kPa) liver fibrosis. Area under the curves (AUC) with 95%-confidence intervals were calculated. Cutoff values were chosen based on Youden’s index. To directly evaluate the performance of different fibrosis scores, we performed a McNemar test to compare CRL-R with the other three fibrosis (LIMV-, LIMA- and LIMVA-FS) to predict significant fibrosis by combining pairs of true (t) and false (f) test results (tt/tf/ft/ff).

To mitigate selection bias, prevalence was estimated in the screened total population (n = 1534) by applying the cutoff values with sensitivities and specificities calculated in the MR elastography sub-group. Positive predictive values (PPV) and negative predictive values (NPV) were then calculated.

For interrater reliability, two blinded radiologists with 5 (V.O.) and 7 (A.H.) years of experience in liver imaging each calculated the CT fibrosis scores in all MR elastography patients (n = 141). Two-way consistency intraclass correlation (ICC) was then calculated and classified as follows: ICC 0.4–0.59 as fair, 0.6–0.74 as good and 0.75–1.00 as excellent [34]. In case of disagreement a consensus reading was performed.

## Results

### Patient characteristics

Patient characteristics of the MR elastography population are shown in Table 1. Patients with elevated liver stiffness indicating liver fibrosis were older (mean age 59±13 vs. 52±13 years, p = 0.012) with a higher proportion of male patients (81% vs. 48%, p = 0.001) than those with normal liver stiffness. Between the patients with beginning and significant liver fibrosis, no significant differences in age were evident (59±13 vs. 58±10 years, p = 0.490). Patients with liver fibrosis had significantly higher levels of liver enzymes (AST 43±26 vs. 25±12, p<0.001, ALT 43±33 vs. 31±37, p = 0.006, GGT 100±124 vs. 31±31, p<0.001) and bilirubin (19±17 vs. 9.2±6, p = 0.003) than patients with normal liver stiffness. The Quick value was reduced (81±20 vs. 98±4, p<0.001), especially in patients with significant liver remodeling (75±20).

**Table 1:** Patient characteristics of the MR elastography study population.

	Normal liver stiffness (n = 105)	Any degree of liver fibrosis (shear modulus ≥ 2.8 kPa)(n = 36)	p—value	Beginning liver fibrosis(shear modulus 2.8–3.5 kPa)(n = 17)	Significant liver fibrosis(shear modulus ≥ 3.5 kPa)(n = 19)	p—value
Age, years	52 ± 13	59 ± 13	0.012	59 ± 15	58 ± 10	0.490
Male, %	50 (48%)	29 (81%)	0.001	13 (76%)	16 (84%)	0.684
BMI, kg/m^2^	27 ± 9	29 ± 7	0.012	29 ± 7	29 ± 7	0.775
Tobacco	16 (15%)	15 (42%)	0.002	4 (24%)	11 (58%)	0.049
Arterial hypertension	22 (21%)	13 (36%)	0.078	4 (24%)	9 (47%)	0.177
Dyslipidemia	8 (11%)	8 (26%)	0.082	5 (29%)	3 (16%)	0.434
Diabetes	5 (5%)	10 (28%)	<0.001	3 (18%)	7 (37%)	0.274
Chronic renal insufficiency	1 (1%)	1 (3%)	0.447	0 (0%)	1 (5%)	0.999
≥ 1 medicament daily	26 (25%)	19 (53%)	0.003	4 (24%)	15 (79%)	0.001
≥ 2 medicaments daily	7 (7%)	13 (36%)	<0.001	2 (12%)	11 (58%)	0.006
AST, U/l	25 ± 12	43 ± 26	<0.001	34 ± 25	50 ± 26	0.032
ALT, U/l	31 ± 37	43 ± 33	0.006	39 ± 29	46 ± 36	0.436
GGT, U/l	31 ± 31	100 ± 124	<0.001	38 ± 20	150 ± 150	<0.001
Alkaline phosphatase, U/l	74 ± 34	87 ± 46	0.184	69 ± 27	102 ± 53	0.180
Bilirubin, μmol/l	9.2 ± 6	19 ± 17	0.003	8 ± 4	25 ± 17	0.001
Albumin	35 ± 7	34 ± 4	0.213	33 ± 4	35 ± 4	0.413
Quick, %	98 ± 4	81 ± 20	<0.001	93 ± 0.3	75 ± 20	0.021
Creatinine, μmol/l	80 ± 22	78 ± 20	0.746	91 ± 19	75 ± 21	0.153

Values are the mean ± SD or n. p-values were calculated using Mann-Whitney U or Fisher’s exact test, as appropriate.

MR = magnetic resonance; BMI = body mass index; AST = aspartate transaminase; ALT = alanine aminotransferase; GGT = gamma-glutamyltransferase

**Fig 4:**
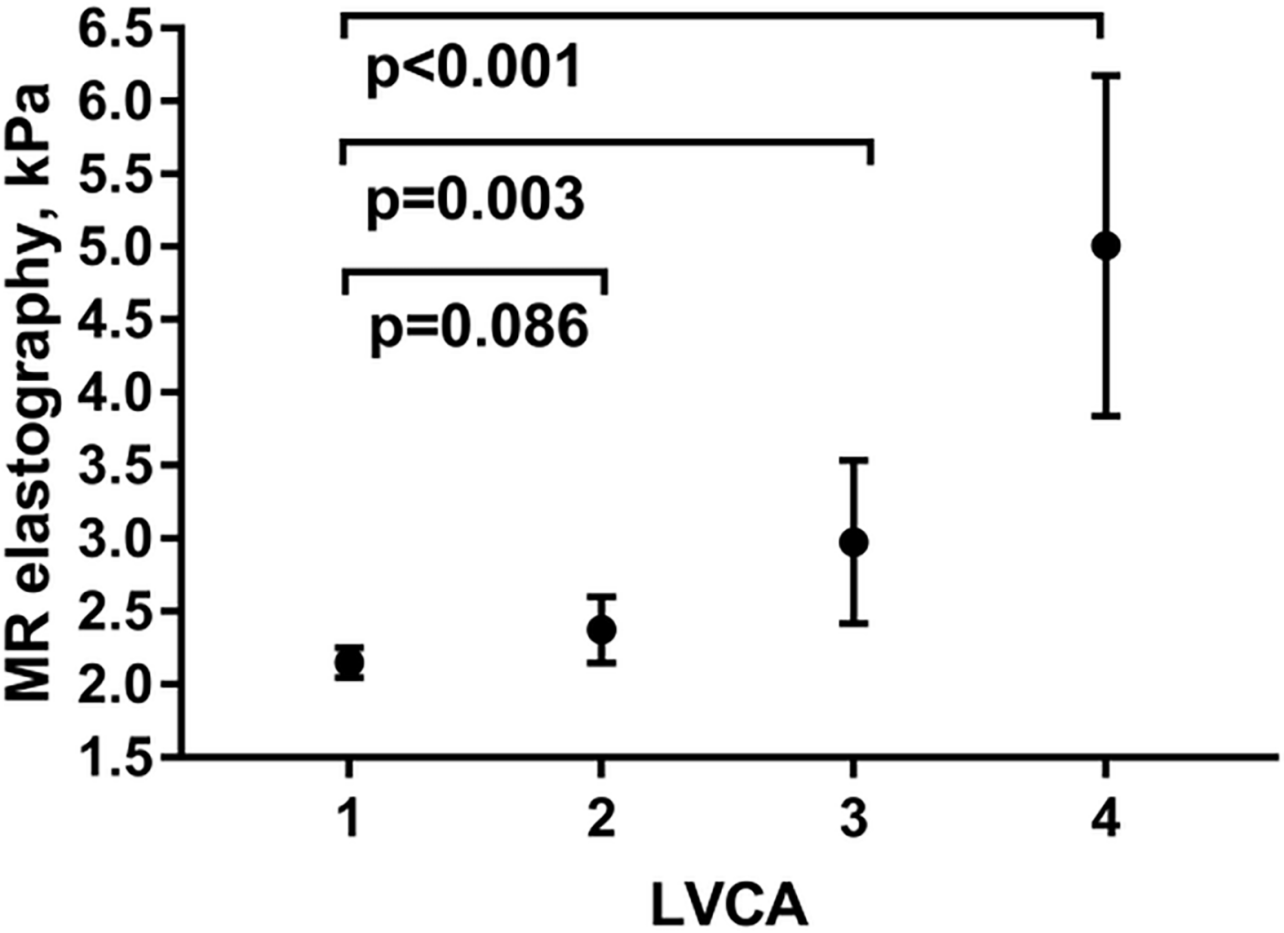
Comparison of LVCA and liver stiffness. Mean liver stiffness as well as 95%-confidence interval are shown for the different LVCA scores (1–4). Comparison between normal LVCA (1) and abnormal LVCA (2–4) was performed using a Mann-Whitney-U test and p-values are indicated. LVCA was defined as liver vein attenuation 1 cm before the confluent compared to inferior cava vein 1 cm before the confluent as follows: 1 hyper-attenuating, 2 iso-attenuating, 3 hypo-attenuating, while 4 indicates no contrast enhancement of liver veins. MR = magnetic resonance; LVCA = liver vein to cava attenuation.

As shown in Table 2, all CT fibrosis scores except CRL-R allowed to significantly differentiate between patients with normal liver stiffness and fibrosis, as well as between patients with beginning and significant liver fibrosis (p<0.001). Hepatic proton density fat fraction, post-viral hepatitis status, chronic viral hepatitis and alcohol units per day were significantly increased in patients with fibrosis compared with patients with normal liver stiffness (p = 0.009, <0.001, 0.001 and <0.001, respectively), while the aspartate-aminotransferase-to-platelet ratio index, chronic hepatitis and alcohol units per day allowed discrimination of patients with beginning and significant liver remodeling (p = 0.006, 0.008 and 0.007, respectively). LVCA correlated well with liver enzymes (AST) as well as fibrosis grade as measured by MRE (r = 0.73, p<0.001, Fig 4).

**Table 2:** Fibrosis scores and specific fibrosis risk factors of the MR elastography study population.

	Normal liver stiffness(n = 105)	Any degree of liver fibrosis (shear modulus ≥ 2.8 kPa) (n = 36)	p—value	Beginning liver fibrosis(shear modulus 2.8–3.5 kPa)(n = 17)	Significant liver fibrosis(shear modulus ≥ 3.5 kPa)(n = 19)	p—value
LIMVA-FS	25 ± 11	12 ± 9	<0.001	18 ± 9	6 ± 3	<0.001
LIMA-FS	1.2 ± 0.6	2.7 ± 1.4	<0.001	1.6 ± 0.8	3.7 ± 1.2	<0.001
LIMV-FS	30 ± 8	21 ± 6	<0.001	24 ± 5	18 ± 5	<0.001
CRL-R	0.85 ± 0.14	1.04 ± 0.20	<0.001	0.99 ± 0.21	1.08 ± 0.18	0.178
APRI	0.7 ± 1.4	1.2 ± 1.2	0.062	0.4 ± 0.3	1.9 ± 1.2	0.006
Hepatic proton density fat fraction, %	9 ± 6	13 ± 10	0.009	13 ± 9	13 ± 11	0.917
Post-viral hepatitis status	3 (3%)	11 (31%)	<0.001	3 (18%)	8 (42%)	0.156
Chronic hepatitis B or C	2 (2%)	7 (19%)	0.001	0 (0%)	7 (37%)	0.008
Ascites	2 (2%)	2 (6%)	0.269	0 (0%)	2 (11%)	0.487
Alcohol units per day	0.0 ± 0.3	0.8 ± 1.9	<0.001	0.4 ± 1.5	1.2 ± 2.2	0.007

Values are mean ± SD or n. p-values calculated using Mann-Whitney U or Fisher’s exact test, as appropriate.

MR = magnetic resonance; LIMVA-FS = liver imaging morphology, vein diameter and attenuation fibrosis score; LIMA-FS = liver imaging morphology and attenuation fibrosis score, LIMV-FS = liver imaging morphology and vein diameter fibrosis score; CRL-R = caudate-right-lobe ratio; APRI = aspartate aminotransferase-to-platelet ratio index

### ROC analysis

**Fig 5:**
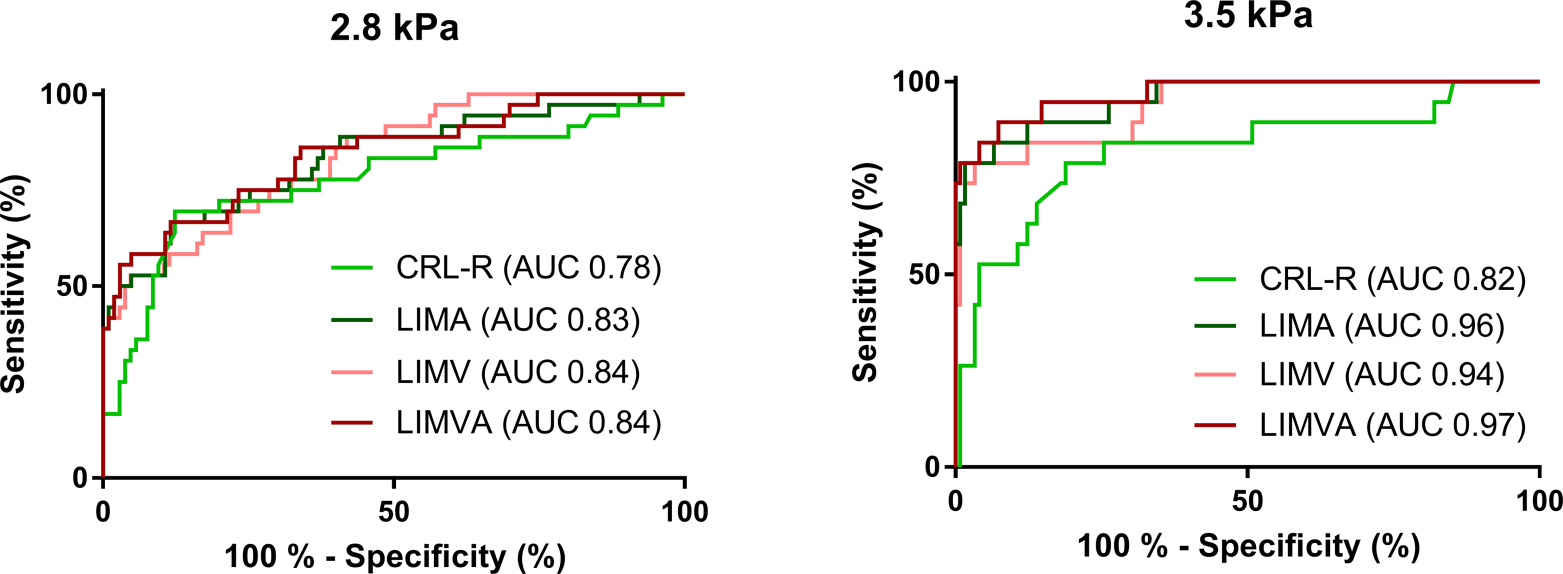
ROC analysis. ROC curves that separate patients with normal liver stiffness from patients with any degree of fibrosis (shear modulus ≥2.8 kPa) are shown in the left panel. ROC-curves of different CT fibrosis scores to predict clinically significant liver fibrosis (shear modulus ≥3.5 kPa) are shown in the right panel. CRL-R = caudate-right-lobe ratio; LIMA-FS = liver imaging morphology and attenuation fibrosis score; LIMV-FS = liver imaging morphology and vein diameter fibrosis score; LIMVA-FS = liver imaging morphology, vein diameter and attenuation fibrosis score.

In the ROC analysis, all measured fibrosis scores revealed similar performances toward differentiating patients with any degree of liver fibrosis from those without fibrosis (AUC 0.78–0.84), as shown in Fig 5. In contrast, attenuation-enhanced LIMA-FS (AUC 0.96, 95%-CI 0.91–1.00) and LIMVA-FS (AUC 0.97, 95%-CI 0.93–1.00) enabled a much better prediction of significant liver fibrosis (corresponding to fibrosis grade F2 or higher) than CRL-R (AUC 0.82, 95%-CI 0.70–0.94), while LIMV-FS showed good performance as well (AUC 0.94, 95%-CI 0.88–0.99). McNemar test showed that CRL-R is significantly inferiority to predict significant liver fibrosis compared to LIMV-FS, LIMA-FS and LIMVA-FS (p = 0.0013, p = 0.0002 and p = 0.0002, respectively). In contrary, LIMV-FS was not significantly inferior to LIMA-FS and LIMVA-FS (p = 0.4497 and p = 0.2278).

Optimal cutoff values with sensitivities, specificities and accuracies are shown in Table 3. The highest PPV to predict significant liver fibrosis of 82% (74–91%) was achieved with a LIMVA-FS cutoff value ≤6.7, while the second best parameter, LIMA-FS, achieved a PPV of 76% (67–96%) with a cutoff value ≥2.85. To rule out significant liver fibrosis, NPV was 98% for both LIMA-FS<2.85 and LIMVA-FS>6.7 but increased to nearly 100% using more conservative cutoff values (LIMA-FS <1.96 and LIMVA-FS<11.7). Extrapolation of the ROC results of the MR elastography study population to 1534 consecutive CT scans is shown in Table 4.

**Table 3:** ROC analysis of liver imaging morphology-based fibrosis scores of the MR elastography study population (n = 141).

	CRL-R	LIMA	LIMA	LIMV	LIMVA	LIMVA
Cutoff value	≥0.98	≥1.96	≥2.85	≤20.0	≤11.7	≤6.7
**Any degree of liver fibrosis** **(>2.8 kPa, equivalent to F1-F4)**						
AUC	0.78 (0.68–0.88)	0.83 (0.75–0.92)	0.83 (0.75–0.92)	0.84 (0.76–0.91)	0.84 (0.76–0.91)	0.84 (0.76–0.92)
Youden’s index	57.1	55.0	43.5	41.6	55.0	40.7
Sensitivity	69%	67%	44%	44%	67%	42%
Specificity	88%	88%	99%	97%	88%	98.
Accuracy	83%	83%	85%	84%	83%	84%
PPV	57% (48–73%)	57% (49–73%)	92% (89–96%)	78% (72–88%)	57% (49–73%)	83% (78–91%)
NPV	93% (86–95%)	92% (94–100%)	88% (79–92%)	88% (79–91%)	92% (85–94%)	88% (78–91%)
**Significant liver fibrosis** **(>3.5 kPa, equivalent to F2-F4)**						
AUC	0.82 (0.70–0.94)	0.96 (0.91–1.00)	0.96 (0.91–1.00)	0.94 (0.88–0.99)	0.97 (0.93–1.00)	0.97 (0.93–1.00)
Youden’s index	60.1	75.5	77.3	75.7	79.7	78.1
Sensitivity	79%	89%	79%	79%	95%	79%
Specificity	81%	86%	98%	97%	85%	98%
Accuracy	81%	87%	95%	94%	86%	96%
PPV	29% (21–46%)	38% (29–57%)	76% (67–96%)	70% (59–83%)	41% (30–59%)	82% (74–91%)
NPV	97% (95–98%)	99% (99–100%)	98% (96–99%)	98% (96–99%)	100% (100%)	98% (96–99%)

The cutoff values are chosen based on Youden’s index. For LIMA and LIMVA, a second cutoff optimized for specificity is shown. PPV and NPV are corrected with the estimated prevalence in the present study population. Estimated PPV and NPV are calculated based on the mean estimated prevalence, as well as the lowest and highest estimated prevalence (shown in brackets).

ROC = receiver operating characteristics; CRL-R = caudate-right-lobe ratio; LIMA-FS = liver imaging morphology and attenuation fibrosis score, LIMV-FS = liver imaging morphology and vein diameter fibrosis score; LIMVA-FS = liver imaging morphology, vein diameter and attenuation fibrosis score; AUC = area under the receiver operating curve; PPV = positive predictive value; NPV = negative predictive value

**Table 4:** Extrapolation of the ROC results of the MR elastography study population to 1534 consecutive CT scans.

	CRL-R	LIMA	LIMA	LIMV	LIMVA	LIMVA
Cutoff value	≥0.98	≥1.96	≥2.85	≤20.0	≤11.7	≤6.7
**Any degree of liver fibrosis (>2.8 kPa, equivalent to F1-F4)**						
Estimated TP	209	147	116	219	160	90
Estimated TN	1080	1161	1261	1011	1143	1291
Estimated FP	153	153	12	30	151	26
Estimated FN	92	73	145	274	80	127
Estimated prevalence	20%	15%	17%	32%	16%	14%
Mean estimated prevalence	19 ± 7%					
**Significant liver fibrosis (>3.5 kPa, equivalent to F2-F4)**						
Estimated TP	96	83	93	207	109	92
Estimated TN	1146	1229	1381	1230	1223	1393
Estimated FP	266	217	35	42	202	24
Estimated FN	26	5	25	55	0	25
Estimated prevalence	8%	6%	8%	17%	7%	8%
Mean estimated prevalence	9 ± 4%					

Values are n or %. Mean prevalence is shown ± standard deviation.

MR = magnetic resonance; ROC = receiver operating characteristics; CRL-R = caudate-right-lobe ratio; LIMA-FS = liver imaging morphology and attenuation fibrosis score, LIMV-FS = liver imaging morphology and vein diameter fibrosis score; LIMVA-FS = liver imaging morphology, vein diameter and attenuation fibrosis score; TP = true positives; TN = true negatives; FP = false positives; FN = false negatives; PPV = positive predictive value; NPV = negative predictive value

### Pearson correlation

**Fig 6:**
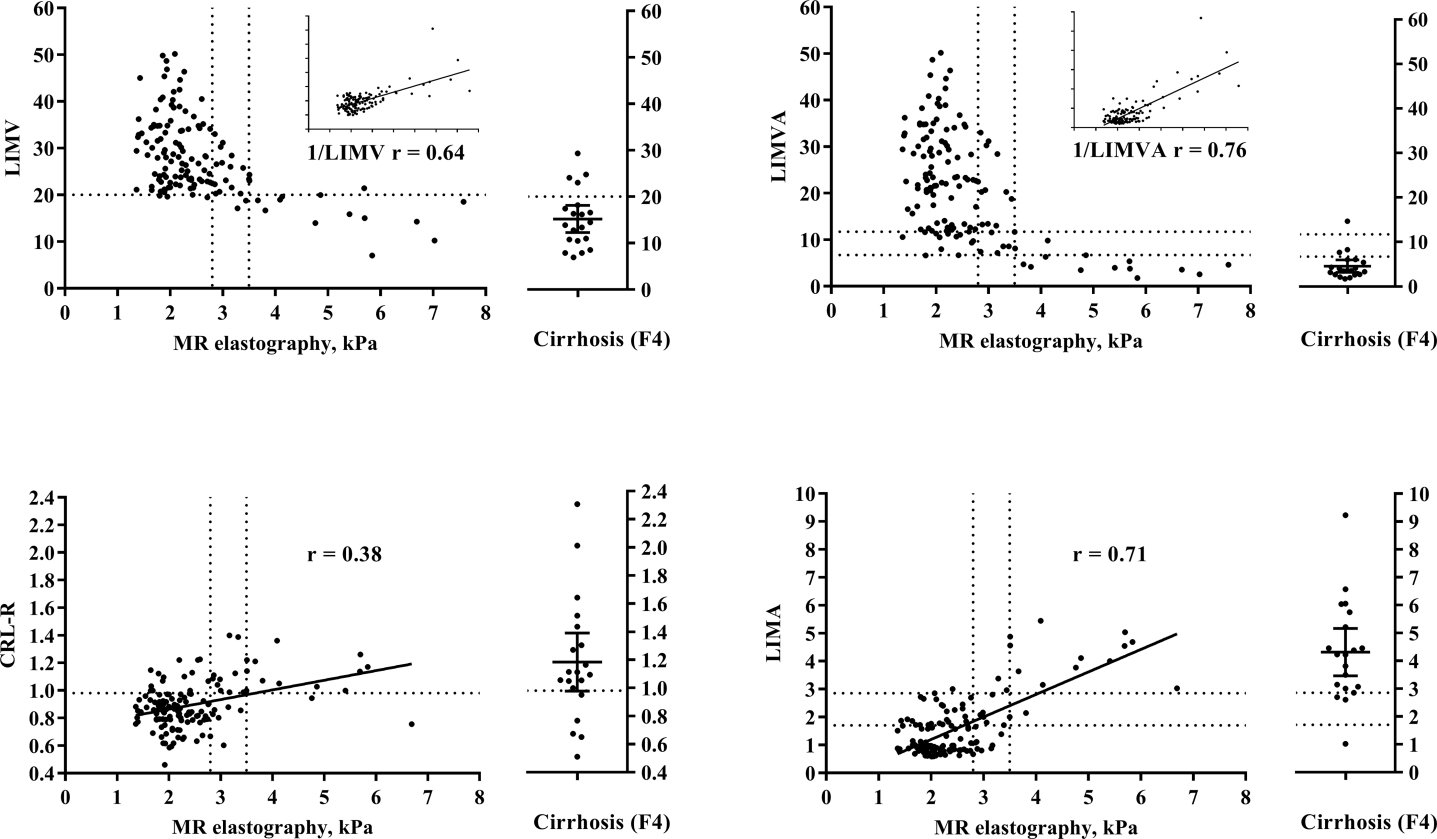
Scatterplots of MR elastography compared with LIMV-FS and CRL-R and their attenuation based complements LIMVA-FS and LIMA-FS. Dashed lines indicate the calculated cutoff values from the ROC analysis on the y-axis and MR elastography cutoff values for early and significant liver fibrosis on the x-axis. On the right side of each figure, 20 consecutive patients with abdominal CT scans and known biopsy-confirmed liver cirrhosis (Metavir score F4) are shown. These patients are shown as positive controls but not included in any statistical analysis. The Pearson correlation r value is shown on every figure, using 1/LIMV-FS and 1/LIMVA-FS due to the inverse correlation with MR elastography. MR = magnetic resonance; LIMV-FS = liver imaging morphology and vein diameter fibrosis score; LIMVA-FS = liver imaging morphology, vein diameter and attenuation fibrosis score; CRL-R = caudate-right-lobe ratio; LIMA-FS = liver imaging morphology and attenuation fibrosis score; ROC = receiver operating characteristics.

When the patients were not separated into categorical subgroups, an increase in liver stiffness correlated best with an increase in 1/LIMVA-FS (r = 0.76, p<0.001), as shown in Fig 6, followed by LIMA-FS (r = 0.71, p<0.001) and 1/LIMV-FS (r = 0.64, p<0.001), while CRL-R (r = 0.38, p<0.001) showed an inferior performance.

### Clinical workup

Of the 141 patients who accepted the invitation for MR elastography, LIMVA-FS cutoff value to predict significant liver fibrosis was positive in 17 patients. Fifteen of these 17 patients had liver fibrosis confirmed by MR elastography. Of note, MR elastography was in 100% accordance with the clinical workup in all patients with significant liver fibrosis. 2 out of 17 patients had a false-positive LIMVA-FS. Another 4 patients were positive for MR elastography with a false-negative LIMVA-FS. From these 4 patients, one had an increased shear modulus of 4.1 kPa and significant liver fibrosis confirmed in the hepatological workup. The three others all had borderline MR elastography values between 3.5–3.54 kPa with clearly elevated liver proton density fat fractions between 32–39% indicating significant liver steatosis. One of these patients underwent liver biopsy with fibrosis Metavir grade F1 and macrovesicular steatosis in 50% of the hepatocytes. Unfortunately, the two other patients did not show up for the hepatological workup despite several invitations.

### Interreader reliability

Interreader reliability was good for all the measured CT fibrosis scores. Two-way consistency ICC was 0.72 for LIMA-FS, 0.72 for CRL-R, 0.65 for LIMV-FS and 0.66 for LIMVA-FS between two independent, blinded readers (V.O. and A.H.).

## Discussion

This study shows that CT-based quantitative scores allow prediction of significant liver fibrosis. Notably, enhancement of these scores with liver vein attenuation in portal venous phase (LIMVA-FS and LIMA-FS), as a simplified surrogate for liver perfusion resulted in a better performance than the purely morphology-based score CRL-R [20,21]. Although we performed the easy to use LVCA on portal venous CT scans and did not use dynamic contrast enhanced CT perfusion studies, this is in accordance with the results by Ronot et al. They have analyzed reduced perfusion in liver fibrosis with CT [26]. Our results are also in agreement with the underlying hypothesis of MR intravoxel incoherent motion (IVIM) [35]. Increased transfer transit time of low-molecular-weight compounds such as contrast agents might be explained by an increasing number of stellate cells, a loss of fenestrae of the sinusoids and the deposition of collagen fibers in the space of Disse in liver fibrosis [36].

All four presented fibrosis scores have the great advantage to allow retrospective calculation on any portal venous abdominal CT scan on axial planes without time-consuming post-processing. Compared with visual analysis on CT scans by experienced radiologists [16], they have all the advantages of quantitative scores that are easily measurable and reproducible.

LIMVA-FS and LIMA-FS, combining morphology and a simplified surrogate for perfusion, showed better performances than pure perfusion studies, such as the MR perfusion study of Hagiwara et al., who reported an AUC of 0.84 to detect fibrosis ≥F3 [27]. If just CRL-R without perfusion was analyzed, the resulting AUC of 0.82 was only slightly lower than the AUC of 0.86 achieved by Pickhardt et al. with more time-consuming caudate to right lobe volumetry [17]. This difference might be explained by the higher accuracy of volumetry relative to the two-dimensional CRL-R calculation but also by the fact that Pickardt et al. used a cutoff value ≥F3, representing a more advanced fibrosis grade than the cutoff value for significant fibrosis we used in this study. However, if CRL-R was extended by the simplified perfusion metric, we showed a higher AUC of 0.96–0.97 to predict significant liver remodeling using LIMA-FS and LIMVA-FS.

Our CT based prevalence estimation of 9% for significant liver remodeling is very comparable to the prevalence of significant fibrosis at 7.5% in the general population using FibroScan® in cross-sectional studies [12]. The prevalence estimation allowed us to calculate PPV and NPV, thus showing that patients with a LIMVA-FS ≤6.7 could be referred to a hepatology department with a PPV of 82% for significant liver remodeling while NPV for patients with LIMVA-FS above this cutoff would be 98%.

Despite the existence of FibroScan® as a cheap, accurate and reliable non-invasive method to stage liver fibrosis, chronic liver disease is frequent [37] and under recognized [5,6]. Because of the ubiquity of CT in clinical practice, extractions of all possible information from abdominal CT scans are compulsory. For this purpose, LIMVA-FS and LIMA-FS are useful non-invasive imaging biomarkers. Patients with unknown liver disease could be depicted and referred to a hepatology department for further workup. Early detection of these patients might allow personalized treatments during reversible fibrosis stages, before irreversible cirrhosis occurs [7], and as a primary prevention of HCC.

**Fig 7:**
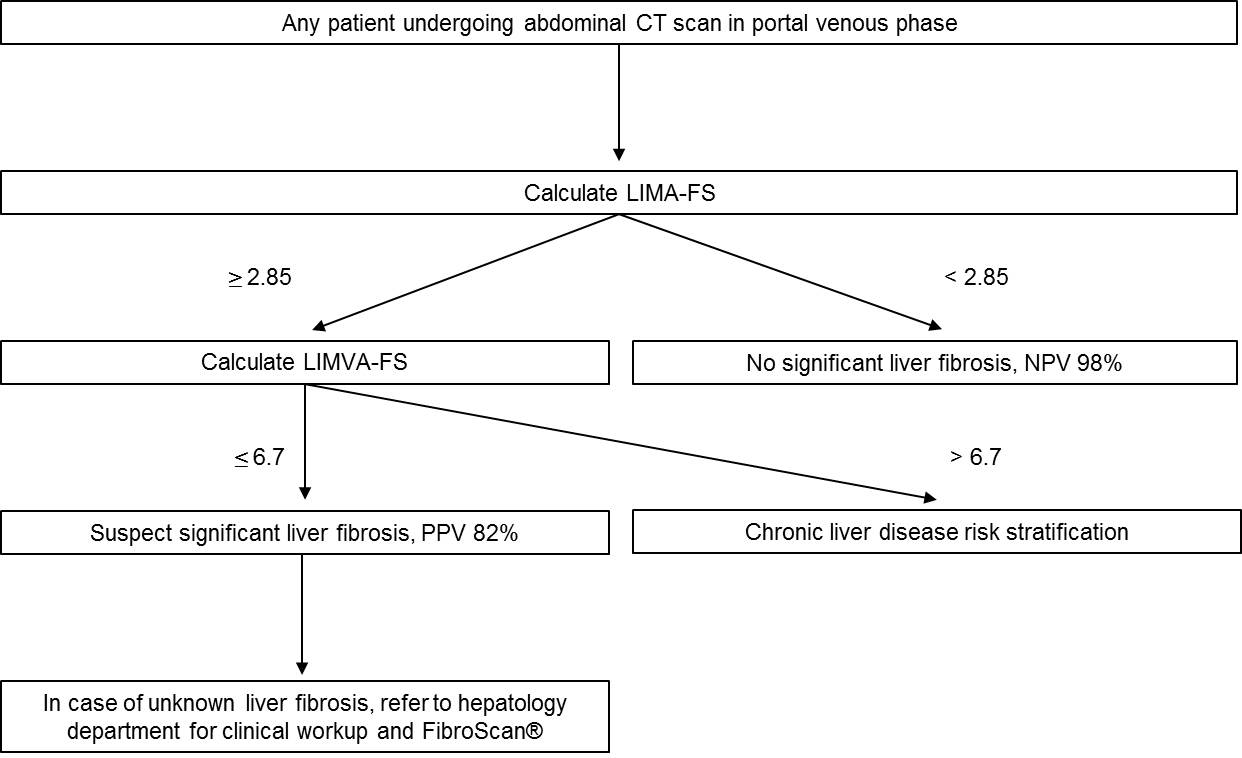
Possible clinical workflow based on LIMA-FS and LIMVA-FS in portal venous abdominal CT scans. CT = computed tomography; LIMA-FS = liver imaging morphology and attenuation fibrosis score; LIMVA-FS = liver imaging morphology, vein diameter and attenuation fibrosis score; NPV = negative predictive value; PPV = positive predictive value.

Based on our results, we propose a possible clinical workflow, as shown in Fig 7. Since LIMA-FS is calculated more quickly than LIMVA-FS, we propose to screen every abdominal CT scan in the portal venous phase with an excellent NPV if LIMA-FS <2.85. This score can be calculated in less than 1 minute on axial CT stacks. In cases of LIMA-FS **≥**2.85, we propose to calculate LIMVA-FS with a PPV of 82% for significant liver remodeling if LIMVA-FS <6.7. In the rare case of a positive LIMA-FS and a negative LIMVA-FS, we recommend a stratification of the hepatic risk factors and consideration of the special case of chronic hepatic congestion due to heart failure, which might present as liver fibrosis with dilated liver veins. In those cases variation of liver veins and IVC enhancement as result of cardiac output and injection properties might influence LIMA-FS and LIMVA-FS.

Our study has several limitations. Due to the ethical considerations, we used MR elastography as the non-invasive gold standard and not liver biopsy to assess liver fibrosis. This allowed us to investigate a general patient population without known chronic liver disease. MR elastography has shown excellent accuracy with biopsy-confirmed fibrosis grades [38,39]. Another possible limitation is that moderately increased liver stiffness may also be caused by hepatic inflammation, steatosis or ingestion of a fatty meal [32]. To mitigate these possible biases, all patients underwent MR elastography in the fasting state, and the hepatic proton density fat fractions were measured, without differences in the hepatic proton density fat fraction between patients with beginning and significant liver remodeling. Another limitation of LIMA-FS and LIMVA-FS involves the normal variants of portal vein anatomy, which are not infrequent. To address this possible drawback, we introduced a definition for the measurement in case of portal venous variants, and interrater reliability was good for all presented CT scores. Last but not least, LVCA may be affected by several confounding factors such as different CT manufacturers and contrast media types and injected flow rates. In addition, LVCA is not a real perfusion parameter since we did not evaluate dynamic contrast enhanced CT perfusion studies but categorized LVCA based on Hounsfield Units in portal venous scans. However, simplified LVCA has the advantage being applicable on any portal venous CT scan of the abdomen without applying higher radiation dose to the patient as it is the case in CT perfusion studies.

In conclusion, notably LIMA-FS and LIMVA-FS, allow to predict significant liver fibrosis with high PPV on routine CT scans. These simple quantifiable metrics are highly reproducible and may be retrospectively calculated on axial planes without time-consuming post-processing. Patients with unknown liver fibrosis may be referred to a hepatology department for specific workup and adequate treatment as a primary prevention of liver cirrhosis, liver failure and HCC.

## Abbreviations

AUC: area under the curve
CRL-R: caudate-right-lobe ratio
CT: computed tomography
CLD: caudate lobe diameter
HCC: hepatocellular carcinoma
LIMA-FS: liver imaging morphology and attenuation fibrosis score
LIMV-FS: liver imaging morphology and vein diameter fibrosis score
LIMVA-FS: liver imaging morphology, vein diameter and attenuation fibrosis score
LVCA: liver vein to cava attenuation
LPV: left portal vein
LVD: liver vein diameter
MR: magnetic resonance
NPV: negative predictive value
PDFF: proton density fat fraction
PPV: positive predictive value
ROC: receiver operating characteristics
RLD: right lobe diameter
RPV: right portal vein
